# Leg-specific modulation of muscle synergies during Tai Chi heel kick in elite athletes

**DOI:** 10.3389/fphys.2025.1727715

**Published:** 2026-01-08

**Authors:** Jinsong Mo, Feiyue Jing

**Affiliations:** 1 College of Physical Education and Health, Guilin Institute of Information Technology, Guilin, China; 2 College of Physical Education and Health, Guangxi Normal University, Guilin, China

**Keywords:** bilateral, elite athletes, heel-kick, muscle synergies, neuromuscular control, Tai Chi

## Abstract

**Purpose:**

The Tai Chi heel-kick is a slow, high-amplitude single-leg movement that places substantial demands on lower-limb coordination, dynamic balance, and controlled force transfer, and is increasingly incorporated into performance training and rehabilitation programs. This study investigated muscle synergies and activation timing differences between the left and right legs of elite Tai Chi athletes during the heel-kick and explored leg-specific neuromuscular control features by combining synergy analysis with spinal segment statistical parametric mapping (SPM).

**Methods:**

Twelve national-level elite Tai Chi athletes were recruited. Surface electromyography (sEMG) signals were collected from key lower-limb muscles, and muscle synergies were extracted using non-negative matrix factorization (NMF). The center of activation (CoA) was calculated using circular statistics, and SPM was applied to examine activation differences across the L2–S2 spinal segments.

**Results:**

Four to five robust muscle synergies were identified for both legs, showing high spatial similarity; however, activation timing differed, with the left-leg synergies peaking earlier and the right-leg synergies delayed. CoA analysis indicated an overall phase advance in the left leg and a phase delay in the right leg. Spinal SPM further revealed inter-limb differences during the mid-to-late phase of the movement (40%–80%), with right-leg activation concentrated in L4–S1 segments, whereas the left leg exhibited a broader distribution.

**Conclusion:**

During the Tai Chi heel-kick, the left and right legs share similar synergy composition but differ in temporal activation patterns and spinal segmental drive, suggesting leg-specific modulation within an overall conserved modular structure. Characterizing these timing asymmetries in highly trained athletes provides a neuromuscular reference for designing Tai Chi–based training drills targeting single-leg control and side-to-side balance, and offers a potential framework for tailoring lower-limb rehabilitation exercises that require coordinated support and kicking functions.

## Introduction

Bilateral lower-limb movements rely on the central nervous system (CNS) to coordinate many muscles into a small number of task-specific control modules, or muscle synergies, enabling robust performance under changing mechanical constraints (d'Avella et al., 2015; Ivanenko et al., 2006). Across a wide range of locomotor tasks, these synergies act as low-dimensional building blocks that are reused while their spatial weighting and temporal activation are flexibly modulated rather than completely reorganized (d'Avella et al., 2015; Zelik et al., 2014; Hagio et al., 2015). Temporal descriptors of the corresponding motor primitives, such as the center of activity (CoA), summarize when each synergy is most active within a movement cycle and can be computed on a circular time base (Zelik et al., 2014; Oshima et al., 2022). CoA and related timing features systematically shift with changes in speed, gravity, or gait phase transitions and are therefore considered sensitive markers of neuromotor strategies (Zelik et al., 2014; Hagio et al., 2015; Janshen et al., 2017). Work on gait transitions and walking under altered gravity has demonstrated that common synergy sets can be maintained while their timing and amplitudes are adjusted to task demands (Sylos-Labini et al., 2022; Hagio et al., 2015; MacLellan et al., 2014). During stability-threatening perturbations such as slips or recovery steps, leg-specific adjustments in synergy composition and timing further limit the generalization of stability performance across tasks (Hagio et al., 2015; Coscia et al., 2015; J et al., 2024).

In clinical populations, particularly individuals with chronic ankle instability (CAI), synergy analyses reveal a largely preserved number and basic structure of locomotor modules but altered muscle weights and activation timing within selected modules (Zhou et al., 2024; Law and Li, 2022; Li et al., 2023a). For example, CAI patients show retimed synergies and increased reliance on proximal muscles during landing and cutting, interpreted as compensatory strategies to stabilize the injured joint and redistribute impact loads (Zhou et al., 2024; Law and Li, 2022; Li et al., 2023a). Together, these findings indicate that subtle, leg-specific modulation of synergy timing and composition is an important mechanism through which the CNS meets limb-specific mechanical and stability demands (Hagio et al., 2015; Coscia et al., 2015; Zhou et al., 2024). However, most of this work has focused on relatively simple locomotor tasks or on asymmetrical conditions imposed by pathology or experimental perturbations, and much less is known about leg-specific synergy modulation in symmetric-appearing, highly skilled movements where the limbs alternate between supporting and striking roles (Hagio et al., 2015; Coscia et al., 2015; J et al., 2024).

Traditional Tai Chi forms provide a natural human model to address this question, because they emphasize slow, continuous weight shifts, prolonged single-leg support and controlled multi-joint movements while being widely used to enhance balance and gait in older adults and people with lower-limb pathology (Liu, 2022; Huang et al., 2024; Xu et al., 2003). Biomechanical and neuromuscular studies have shown that Tai Chi practice involves substantial lower-limb joint excursions and muscle activation levels comparable to or exceeding those observed during walking, and that long-term practice can favorably modify lower-limb kinetics and activation patterns (Liu, 2022; Wayne et al., 2021). Competitive routines with pronounced single-leg stance phases, such as the 143°D balance movement, impose high demands on dynamic stability, precise control of the center of mass, and coordinated lower-limb muscle recruitment (Li et al., 2023b).

Recent EMG- and synergy-based analyses of specific Tai Chi leg techniques, including the leg stirrup and standardized heel-kick movements, have identified phase-specific muscle synergies that support both fluid segmental coordination and postural stability (Pan et al., 2025; Zhang et al., 2025). Elite Tai Chi athletes, who have typically undergone many years of intensive technical training, exhibit highly consistent kinematic and neuromuscular patterns, so any residual inter-limb differences in synergy organization or timing are more likely to reflect purposeful functional specialization than incomplete skill acquisition (Wayne et al., 2021; Pan et al., 2025; Zhang et al., 2025).

Within this context, the standardized heel-kick (Deng Yi Gen) in competition Tai Chi is an ideal model task for probing bilateral neuromuscular control, as it combines prolonged single-leg support, rapid heel acceleration and precise endpoint placement with stringent requirements on trunk alignment and whole-body balance (Wayne et al., 2021; Li et al., 2023b; Zhang et al., 2025). During this movement, the supporting leg must generate and modulate forces to maintain dynamic postural stability, whereas the kicking leg must accurately direct the distal segment in space, raising the question of whether these distinct functional roles are reflected in leg-specific modular timing strategies despite the movement’s symmetric visual appearance.

Therefore, the present study applied muscle synergy analysis of multi-muscle surface electromyography (sEMG) to investigate bilateral neuromuscular control during the standardized heel-kick in elite Tai Chi athletes. Our primary objective was to determine whether the heel-kick is governed by a common set of lower-limb synergies in both legs and whether the temporal deployment of these synergies—quantified by the CoA of their motor primitives—differs systematically between the supporting and kicking limbs. We hypothesized that the basic modular organization would be similar across legs, reflecting shared task requirements, but that the timing of synergies related to propulsion and postural stabilization would exhibit leg-specific CoA shifts, indicating distinct temporal strategies for force transmission and balance control in elite performers.

## Materials and methods

### Participants

A total of 12 high-level Tai Chi athletes (all male) were recruited for this study, including 6 National Master Sportsmen and 6 First-Class athletes. The participants had a mean age of 18.83 ± 3.32 years, a mean height of 173.08 ± 5.58 cm, and a mean body mass of 63.33 ± 6.56 kg, with an average of 8.08 ± 3.78 years of Tai Chi training experience. All participants possessed good physical fitness and athletic ability and reported no musculoskeletal injuries or disorders within the previous year. After receiving a detailed explanation of the purpose of the study, procedures, and potential risks, all participants provided written informed consent. This study was approved by the Human Research Ethics Committee of Guangxi Normal University (Approval No. 20251015001).

### Experimental setup and data acquisition

During the heel-kick trials, ground reaction force (GRF) data were collected using a force platform (Kistler, 9260AA6, Switzerland) at a sampling frequency of 1,000 Hz. Surface electromyography (sEMG) signals were recorded from twelve bilateral muscles: Rectus Femoris (RF), Vastus Lateralis (VL), Tibialis Anterior (TA), Biceps Femoris (BF), Medial Gastrocnemius (MG), and Gluteus Maximus (GMax). Prior to electrode placement, the skin was shaved, lightly abraded, and cleaned with alcohol to reduce impedance.

Kinematic data were acquired using a three-dimensional optical motion capture system (Vicon T40, Vicon Motion Systems Ltd., UK) with ten cameras operating at 200 Hz. Retro-reflective markers were attached to anatomical landmarks based on published protocols (Dumas et al., 2007; Kipp et al., 2012), yielding a full-body model with 38 marker points. The placement scheme included: forehead (LHAD, RHAD), occipital bone (LFAD, RFAD), sternum (CLAV, STRN), C7, T10, anterior superior iliac spines (LASIS, RASIS), iliac crests (LICST, RICST), posterior superior iliac spines (LPSIS, RPSIS), greater trochanters (LTROC, RTROC), thigh tracking clusters (LTH1, LTH2; RTH1, RTH2), medial and lateral femoral condyles (LMEP, LLEP; RMEP, RLEP), shank tracking clusters (LSK1, LSK2; RSK1, RSK2), medial and lateral malleoli (LMME, LLME; RMME, RLME), calcanei (LHEEL, RHEEL), first metatarsal heads (LHM1, RHM1), and fifth metatarsal heads (LHM5, RHM5).

### Testing procedure

Participants were informed of the experimental procedures and safety considerations before signing the consent form. Anthropometric measurements were obtained, including segment lengths of the trunk, upper arms, forearms, thighs, shanks, and feet. Markers were securely affixed according to the Visual3D full-body model, and participants wore laboratory-standard tight-fitting garments to reduce marker movement errors. All markers were attached by the same examiner to ensure consistency. After placement, each sensor and electrode connection were carefully checked. The skin was additionally cleaned with 75% alcohol wipes, and excess hair was removed to ensure high-quality EMG signals. Maximum voluntary contraction (MVC) trials were collected for each target muscle.

Before the formal trials, a static calibration trial was recorded, followed by a 5-min warm-up to prevent muscle strain during testing. For data collection, participants were instructed to stand within the capture volume, with each foot placed on a separate force platform. Under the examiner’s verbal command, participants performed the standardized Chen-style Tai Chi heel-kick movement. All trials were video recorded for verification.

**FIGURE 1:**
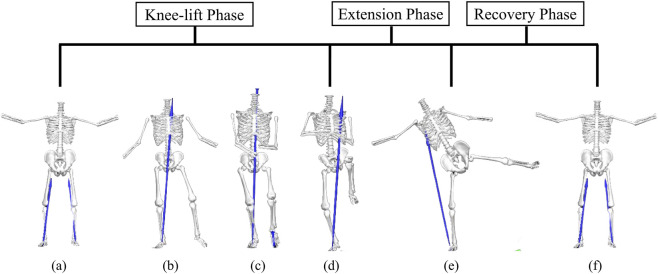
Phase segmentation of the Tai Chi heel-kick. **(a)** initial posture; **(b)** foot-off; **(c)** empty-stance configuration; **(d)** maximal knee flexion; **(e)** maximal kicking extension; **(f)** stabilized (return-to-neutral) posture.

The heel-kick movement cycle was defined as the interval from ipsilateral heel-off to the return of the swing leg to the neutral position. The start of the cycle was objectively identified by heel-off (vertical GRF <20 N), and the end was defined by the kinematic zero-crossing at which the key joint angles of the swing limb returned to neutral (or by the foot returning to its initial pose). Within this interval, three phases were delineated: (i) Knee-lift Phase (Figures 1a–d), characterized by hip flexion and knee flexion elevating the limb and terminating at the peak knee flexion; (ii) Extension Phase (Figures 1d,e), during which knee extension together with ankle plantarflexion rapidly projects the shank and foot forward, ending at the peak heel (or toe) linear velocity or the maximal forward reach; and (iii) Recovery Phase (Figures 1e,f), in which the swing limb retracts along the original path and returns to neutral, thereby completing the cycle. All cycles were time-normalized to 0%–100% (200 equally spaced samples), and event detection was automated and verified against video and marker trajectories.

### EMG signal processing

Raw EMG signals were sampled at 1,000 Hz and processed using a zero-lag, fourth-order Butterworth filter. First, the signals were high-pass filtered at 20 Hz to attenuate movement artefacts and low-frequency baseline drift while preserving the main power spectrum of myoelectric activity associated with dynamic lower-limb tasks. This cut-off lies within the 20–50 Hz range that has been systematically evaluated in locomotor synergy studies, where high-pass filters at or above 20 Hz have been shown to exert only minor effects on the spatial structure of extracted synergies when applied consistently across conditions (Santuz et al., 2017a; Banks et al., 2017). After full-wave rectification, the EMG was low-pass filtered at 10 Hz to obtain a linear envelope that reflects the underlying neural drive but remains sufficiently smooth for robust NMF-based synergy extraction. Cut-off frequencies between approximately 5 and 20 Hz are widely used for constructing EMG envelopes in gait and locomotor synergy analyses, and both methodological work and empirical studies have indicated that low-pass cut-offs around 8–12 Hz provide a good compromise between temporal resolution and signal-to-noise ratio (Santuz et al., 2017a; Banks et al., 2017; Shiavi et al., 1998). In line with these guidelines, the combination of a 20 Hz high-pass filter and a 10 Hz low-pass filter in the present study represents a trade-off between removing low-frequency artefacts and avoiding over-smoothing of the temporal activation patterns. For normalization, the peak EMG amplitude of each muscle across all heel-kick trials was used as the reference, and each trial was expressed as a percentage of this peak. The normalized envelopes were then time-normalized to 200 points across the heel-kick cycle by linear interpolation.

### Muscle synergy extraction

We employed non-negative matrix factorization (NMF) to extract muscle synergies from the EMG data of each subject and each trial (Lee and Seung, 1999), Iterative updates were performed until the reconstruction coefficient of determination (*R* ^2^) converged (Tresch et al., 2006; Cheung et al., 2005). The algorithm uses iterative multiplicative update rules to decompose muscle activity (*D(t)*) into time-invariant synergy vectors (*Wi*), scaled by time-varying activation coefficients (*Ci(t)*) as follows (Equations 1–3):

$$D\left(t\right)\approx \sum_{i=1}^{N_{syn}}\;C_{i}\left(t\right)W_{i}$$ (1)

$$R^{2}=1-\frac{SSE}{SST}$$ (2)

$$\text{SST}=\sum_{i,j}{\left(D_{ij}-mD_{i}\right)}^{2},\text{SSE}=\sum_{i,j}{\left(D_{ij}-{\left[WC\right]}_{ij}\right)}^{2}$$ (3)

where *D(t)* denotes the muscle activity time series, *Ci(t)* the time-varying activation coefficients, *Wi* the time-invariant synergy vectors, *R* ^*2*^ the goodness-of-fit, *SST* the total sum of squares, *SSE* the sum of squared errors, *Nsyn* the number of extracted synergies, *i* the time point, *j* the muscle channel, and *N* the number of synergies extracted per trial.

Since 12 muscles were recorded, a maximum of 12 synergies could be extracted. The number of synergies was defined as the minimum number required to reach approximately 80% EMG reconstruction *R* ^2^, and the solution yielding the highest *R* ^2^ was selected for further analysis. The calculation of *R* ^2^ followed Equation 2. Each extraction was repeated 20 times, and the iteration was terminated when the change in EMG reconstruction *R* ^2^ was <0.001% over 20 consecutive iterations (Cheung et al., 2012).

To characterize differences in synergy vectors across conditions, k-means clustering was applied to identify representative synergy vectors for each group (Hartigan and Wong, 1979). The MATLAB k-means function was used, with initial cluster centroids randomly selected from the set of synergy vectors. Clustering was repeated 1,000 times with different initial centroid estimates, and the solution with the smallest total distance to the centroids was retained. The number of clusters was determined using the gap statistics. A reference dataset (N = 400) was generated by uniformly sampling within the bounds of the original synergy dataset. Each reference dataset was clustered using k-means (100 repetitions) for cluster numbers ranging from 2 to 20. The optimal number of clusters, k, was defined as the smallest satisfying k (Equation 4):

$$\text{Gap}\;\left(k\right)\geq \text{Gap}\;\left(k+1\right)-\text{sd}\;\left(k+1\right)$$ (4)

where *Gap(k)* is the gap statistic for *k* clusters, and *sd(k)* is the standard deviation of clustering compactness in the reference dataset. Extracted synergies were then sorted by cosine similarity (Coscia et al., 2011; Torres-Oviedo and Ting, 2007)。Each subject’s synergy vectors (W and C) were compared to reference patterns, and the pair with the highest similarity was assigned to the same class. In cases of ambiguity, the synergy was classified based on the highest similarity and confirmed by visual inspection to avoid misclassification.

### Muscle synergy evaluation

Circular statistics were applied to compute the center of activity (CoA) of each synergy’s activation coefficient (Berens, 2009), and polar plots were generated to illustrate the relative timing of activations (Equations 5–7).

$$A=\sum_{t=1}^{N}\left(\cos\theta_{t}\times {Act}_{t}\right)$$ (5)

$$B=\sum_{t=1}^{N}\left(\sin\theta_{t}\times {Act}_{t}\right)$$ (6)

$$CoA=\tan^{-1}\left(\frac{B}{A}\right)$$ (7)

Specifically, each time point of the movement cycle (0%–100%) was mapped to an angle (0°–360°), denoted as $\theta_{t}$ , while ${Act}_{t}$ represented the activation magnitude at that time point. The CoA was defined as the angular direction of the centroid vector in polar coordinates (first trigonometric moment).

### Spinal segmental output

To characterize the spatiotemporal organization of the overall motor output, ensemble-averaged EMG envelopes were mapped onto the approximate rostrocaudal locations of the lumbosacral motoneuron (MN) pools to reconstruct spinal segmental motor output. The mapping procedure followed previous spinal map studies by Ivanenko and colleagues and La Scaleia et al., in which EMG activity is projected onto spinal segments between L2 and S2 using anatomical myotomal charts and histological MN counts (Ivanenko et al., 2007; Ivanenko et al., 2006; Ivanenko et al., 2008; Ivanenko et al., 2013).

Segment–muscle weighting coefficients $k_{ji}$ were derived from standard myotomal charts (Kendall et al., 1994) and their implementation in spinal mapping studies. For each of the twelve recorded muscles, we identified the lumbar and sacral segments reported to contribute to its innervation within L2–S2. Specifically, rectus femoris and vastus lateralis were associated with L2–L4, tibialis anterior with L4–L5, biceps femoris with L5–S2, medial gastrocnemius with S1–S2, and gluteus maximus with L4–S1. For each muscle i , $k_{ji}$ was set to 1 for all segments j that innervate that muscle and 0 otherwise, and $l_{i}$ was defined as the total number of innervating segments for that muscle. In this way, the term $k_{ji}/l_{i}$ distributes the activity of muscle i equally across its contributing segments, while $m_{j}$ denotes the number of muscles innervated by segment j .

Segment-specific motoneuron pool sizes $MN_{j}$ were taken from classic histological counts of limb α-motoneurons in individual lumbosacral segments (L2–S2) reported by Tomlinson and co-workers (Tomlinson et al., 1973; Tomlinson and Irving, 1977; Irving et al., 1974). We used the mean number of MNs per segment across these studies and normalized the values by the maximum MN count so that the reconstructed spinal output reflects the relative size of the segmental motor pools. The motor output pattern of each spinal segment $S_{j}$ over the heel-kick cycle was estimated using the following weighted formula (Equation 8):

$$S_{j}=\frac{\sum_{i=1}^{m_{j}}\left(\frac{k_{ji}}{l_{i}}\cdot {EMG}_{i}\right)}{\sum_{i=1}^{m_{j}}\frac{k_{ji}}{l_{i}}}\cdot {MN}_{j}$$ (8)

where $EMG_{i}$ is the normalized linear envelope of the i -th muscle, $k_{ji}$ is the segment–muscle weighting coefficient, $l_{i}$ is the number of segments innervating muscle i , $m_{j}$ is the number of muscles innervated by segment j , and $MN_{j}$ is the normalized number of motoneurons in segment j . Spatiotemporal rostrocaudal activation patterns (L2–S2 × 0%–100% of the heel-kick cycle) were visualized using filled contour plots to provide a compact representation of the segmental motor output.

### Statistical analysis

Muscle synergy patterns extracted *via* NMF were evaluated using variance accounted for (VAF) and reconstruction accuracy (*R* ^2^). Circular statistics were employed to calculate CoA, and phase differences across conditions were compared using the Watson–Williams test. For spinal segment activation time series, one-dimensional statistical parametric mapping (SPM1D) was applied with paired t-tests, and random field theory (RFT) was used for multiple comparison correction. For non-circular variables (e.g., VAF, synergy strength), the Shapiro–Wilk test was used to assess normality. Data meeting normal distribution assumptions were analyzed with paired or independent-sample t-tests; otherwise, the Mann–Whitney U test or Wilcoxon signed-rank test was used. Effect sizes (Cohen’s d) were reported for all significant results. The significance threshold was set at p < 0.05.

## Results

**FIGURE 2:**
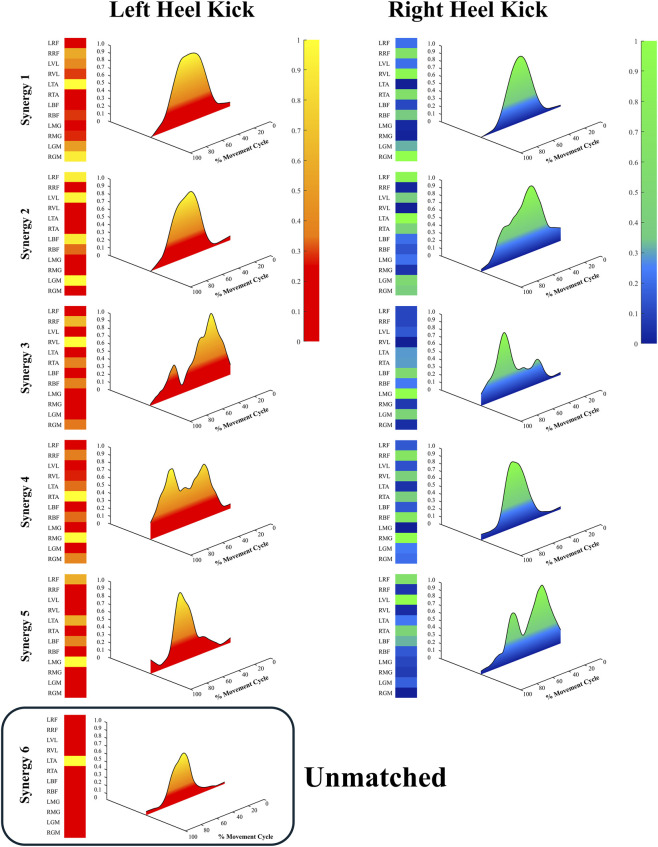
Muscle synergies and activation patterns of the left and right legs. Blue denotes the left leg, red denotes the right leg. Heatmaps represent synergy composition (W), and curves represent activation coefficients (C). The vertical axis indicates activation level, and the horizontal axis indicates normalized time (0%–100%).

Figure 2 illustrates the extracted synergy composition (W) and corresponding activation coefficients (C) of the left and right legs during the heel-kick movement. In both legs, five common synergies (Synergies 1–5) were consistently identified, and an additional synergy was observed only in the left leg (Synergy 6, highlighted as “unmatched” in Figure 2). The five common synergies showed clear left–right pairs with highly similar compositions, indicating that comparable muscle groups were recruited bilaterally to perform the task, whereas the extra left-leg synergy suggests a leg-specific refinement of neuromuscular control during the kicking action.

Synergy 1 was dominated by proximal extensors (rectus femoris, vastus lateralis, and gluteus maximus) and exhibited a broad activation in the early portion of the cycle, spanning the knee-lift and early single-leg support phases. This pattern is consistent with a support/propulsion synergy, contributing to center-of-mass elevation, initial force generation, and stabilization of the hip and knee as the body transitions into single-leg stance. Synergy 2 showed higher weighting of tibialis anterior, with moderate activity of the knee extensors, and peaked during the mid-phase of the cycle when the kicking leg was advancing forward. This synergy therefore appears to function primarily as a limb-guidance synergy, responsible for ankle dorsiflexion, foot orientation, and precise trajectory control of the distal segment.

Synergy 3 was characterized by stronger contributions from biceps femoris together with medial gastrocnemius, with activation concentrated around the late-extension and early-retraction phases. Such a temporal and muscular pattern is compatible with a deceleration/impact-absorption synergy, assisting in braking the rapidly extending shank, modulating plantarflexor output, and ensuring a controlled transition from maximal extension into recovery. Synergy 4, which showed a more tonic activation of gluteus maximus and biceps femoris across the mid-to-late portion of the cycle, is best interpreted as a balance-stabilization synergy, contributing to trunk and pelvic alignment and lateral stability during prolonged single-leg support. In the left leg, Synergy 6 presented an additional burst of activity involving tibialis anterior and proximal muscles near the transition from extension to recovery, suggesting a fine-tuning synergy that may facilitate leg repositioning, anticipatory preparation for weight transfer, or subtle adjustments in end-point control on the dominant kicking side.

The activation patterns (C) revealed that these synergies were recruited in a temporally organized sequence: support/propulsion and balance-stabilization synergies were mainly active during the early and mid-stance portions of the cycle, whereas limb-guidance and deceleration/impact-absorption synergies peaked during mid-to-late phases corresponding to leg extension and retraction. Overall, the presence of five matched synergies in both legs indicates a largely conserved modular structure, while the additional left-leg Synergy 6 and leg-specific differences in the temporal deployment of these modules reflect specialized roles in force generation, balance control, and precise limb guidance during the heel-kick.

**FIGURE 3:**
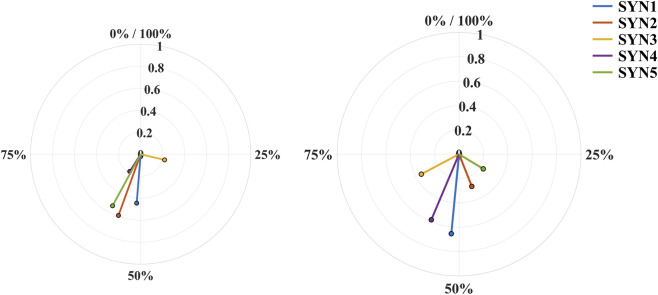
Mean center of activity (CoA) of synergies in the left and right legs. Circular coordinates represent the normalized movement cycle (0–360°). The arrow direction indicates the mean CoA, and the arrow length represents the concentration.

Figure 3 shows the mean center of activity (CoA) and concentration of each synergy for the left and right legs, plotted in polar coordinates across the entire heel-kick cycle. For the support/propulsion synergy (Synergy 1) and the balance-stabilization synergy (Synergy 4), the CoA of both legs was mainly located in the middle portion of the cycle, indicating that proximal extensors and stabilizing muscles were most strongly recruited during single-leg support and redirection of the center of mass. For the limb-guidance and deceleration/impact-absorption synergies (Synergies 2 and 3), the CoA values clustered more in the mid-to-late phase of the cycle, consistent with their respective roles in controlling forward swing of the shank and subsequently braking the kicking leg. All CoA vectors were located within approximately 40%–70% of the normalized cycle. This distribution reflects relatively weak and more dispersed synergy activation in the early preparation and late recovery phases, with clear peaks emerging around mid-cycle. The CoA was calculated over the full 0%–100% time-normalized cycle without applying any amplitude thresholds; therefore, the absence of vectors outside this interval does not imply a complete lack of muscle activation. Notably, some synergies showed leg-specific phase differences. In particular, the CoA of the limb-guidance synergy tended to occur slightly earlier in the left leg than in the right leg, suggesting stronger feedforward activation of ankle dorsiflexion and distal control on the left side.

Conversely, for the deceleration/impact-absorption synergy, the right leg exhibited a slightly later CoA but a higher concentration, indicating a more focused and temporally stable pattern during the terminal extension and retraction phases. In addition, the balance-stabilization synergy showed higher concentration in the supporting leg under each condition, suggesting more stereotyped timing when its primary role is to maintain trunk and pelvic stability. Overall, although the global CoA distribution patterns were similar between legs, subtle differences in phase and concentration at the level of individual functional synergies indicate mild bilateral asymmetries in the temporal strategies used for propulsion, balance stabilization, and limb guidance.

**FIGURE 4:**
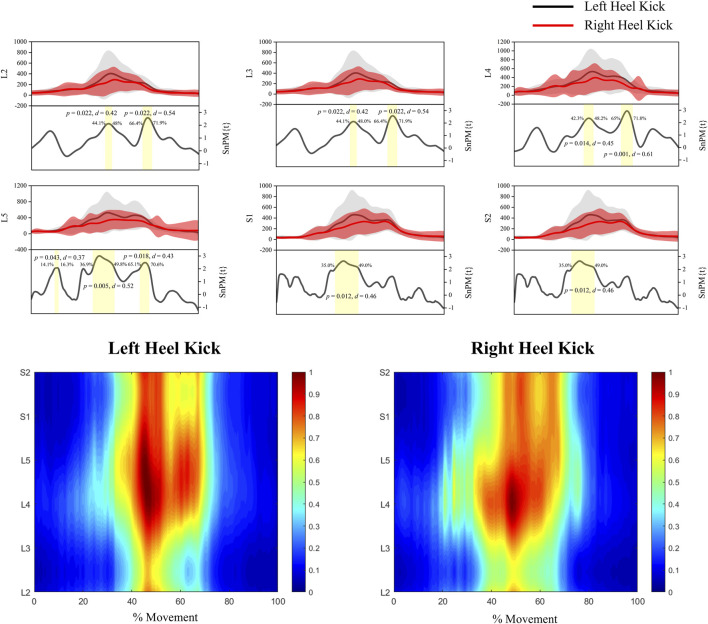
Statistical parametric mapping (SPM) and spinal activation distribution during left- and right-leg heel-kick. Comparison of spinal segments L2–S2 between left-leg kicks (black curves) and right-leg kicks (red curves). Shaded areas indicate standard deviations; yellow regions denote intervals with significant differences (p < 0.05), with corresponding effect sizes (Cohen’s d) (Bottom) Iso-intensity maps of spinal activation (L2–S2) across the full movement cycle (0%–100%) for left-leg (left panel) and right-leg (right panel) kicks. Color gradients represent normalized activation intensity, with red indicating the highest level of activation.

As shown in Figure 4, spinal statistical parametric mapping (SPM) revealed significant lateral differences across multiple segments. Specifically, L2, L3, L4, L5, S1 and S2 showed significant differences between left- and right-leg heel-kicks (p < 0.05), with effect sizes ranging from moderate to large (d = 0.37–0.61). These differences were predominantly observed during the mid-to-late phase of the movement (approximately 40%–80% of the cycle), corresponding to the main activation periods of the support/propulsion, limb-guidance and deceleration/impact-absorption synergies. In contrast, segmental outputs in both legs were close to baseline during the first and last ∼20% of the cycle, which correspond to quiet standing before heel-off and after return to the neutral posture. Segmental time series and iso-intensity maps were computed over the full 0%–100% interval without temporal truncation; the blue bands at the beginning and end of the maps therefore reflect very low normalized amplitudes near the lower end of the colour scale rather than explicit thresholding or removal of data.

Iso-intensity mapping further revealed that, for both left- and right-leg kicks, spinal activation displayed a continuous rostro-caudal distribution from L2 to S2, with the strongest activity occurring during the mid-phase of the movement cycle. However, right-leg kicks exhibited a more concentrated activation pattern, with pronounced peaks at L4–L5 and S1—segments that primarily innervate quadriceps, tibialis anterior and plantarflexor muscles—consistent with a more focused spinal drive to the functional synergies responsible for propulsion and limb guidance. In contrast, left-leg kicks showed a broader distribution covering a wider range of segments, suggesting a more distributed neural drive that may support simultaneous demands for balance stabilization and fine-tuning of the kicking trajectory. In the SPM waveforms (upper panels), mean segmental outputs tended to be slightly higher for left-heel kicks than for right-heel kicks across several segments. This amplitude difference may reflect a combination of the different functional roles of the two legs during the task and long-term training exposure; to provide context, we now report leg dominance, typical initiation side and training symmetry in the Participants section. Given the cross-sectional design, however, the present data do not allow us to attribute these differences unequivocally to training-induced asymmetry. Taken together, these results support the existence of inter-limb differences in spinal-level implementation of functional synergies during the heel-kick.

## Discussion

This study aimed to uncover potential differences in muscle synergies between the two legs of elite Tai Chi athletes during the heel-kick movement. The results demonstrated that although the synergy composition of the left and right legs was highly consistent—each comprising hip extensor–dominant, knee extensor–dominant, ankle dorsiflexor and postural stabilization synergies—their activation timing and spinal drive patterns differed. Specifically, the muscle synergy centers of activity (CoA) of the left leg were overall advanced, indicating earlier involvement during movement initiation and the early force-production phase, whereas the right leg exhibited a delayed trend, with activation more concentrated in the mid-to-late phase of the movement. Moreover, spinal statistical parametric mapping (SPM) revealed significant lateral differences across multiple L2–S2 segments during the mid-to-late phase. Right-leg drive was more concentrated in L4–S1, whereas left-leg activation was distributed more broadly. These findings are consistent with the hypothesis that, while the synergy sets are similar bilaterally, some degree of functional lateralization may exist in temporal control and spinal output patterns during the heel-kick.

Our results align with existing literature on the modular organization of motor control. Numerous studies have shown that complex movements can be generated by combinations of a limited set of muscle synergies, a low-dimensional control strategy that has been validated in contexts such as gait transitions and external perturbations (Liu and Gutierrez-Farewik, 2022). The present study further suggests that even in symmetric, high-load extension tasks, bilateral synergy composition remains highly conserved, while activation timing is flexibly adjusted according to functional demands. This observation is compatible with the framework proposed by Santuz et al., (2017b), emphasizing “stable synergy structures with flexible temporal modulation.” Furthermore, the inter-leg differences in CoA observed in our study are in line with the findings of Oshima et al. (Choi and Bastian, 2007) in split-belt gait experiments, which reported leg-specific variations in the number and temporal features of synergies, reflecting distinct neural control strategies.

Our results also relate to the long-standing notion of asymmetric bilateral control. Previous studies have indicated that even in healthy populations, functional differences can exist between the two legs; the dominant leg tends to assume force-generation tasks, while the non-dominant leg contributes more to stabilization and balance (Carpes et al., 2010; Sadeghi et al., 2000). The observation that the left leg tended to show earlier synergy activation whereas the right leg displayed relatively delayed activation is compatible with such a functional differentiation. However, the present data do not include direct biomechanical measures such as joint moments or center-of-pressure trajectories, so we cannot demonstrate that the left leg was primarily responsible for propulsion and the right leg for stabilization in a strict mechanical sense. Alternative interpretations should therefore be considered. For example, the specific timing pattern may partly reflect technical requirements of the Chen-style routine itself, including the “Twining Force” principle and choreographed sequencing of trunk rotation and limb movements, which might prescribe earlier activation of the kicking leg and a later modulation of the supporting leg, independently of any inherent neurological asymmetry.

From a neural control perspective, advanced or delayed synergy activation can be viewed as one possible indication of differentiated roles of the two legs in motor execution. The earlier activation of the left leg may reflect a tendency for this limb to participate more prominently in movement preparation and early force transmission, whereas the relatively delayed activation of the right leg may be associated with a greater contribution to modulation and stabilization in the mid-to-late force-production phase, consistent with its spinal drive being more concentrated in L4–S1 segments. These interpretations, however, should be regarded as working hypotheses rather than definitive causal statements, and future studies integrating kinetic and kinematic analyses will be required to verify how timing asymmetries relate to propulsion and balance control in this task.

Another plausible explanation for the observed lateral specificity is long-term training-related adaptation. Tai Chi practice emphasizes bilateral, alternating force generation and dynamic balance control, which could, over years of practice, promote the emergence of a relatively stable “left–right division of labor” strategy to achieve efficiency and stability in execution. Similar adaptive adjustments have been reported in developmental studies of motor primitives in children, where functional lateralization emerges early in life (Dominici et al., 2011). Nevertheless, the present cross-sectional study did not include a control group of novice practitioners, nor did it quantify athletes’ detailed training history or any systematic lateral emphasis. Consequently, training-induced adaptation should be regarded as a plausible direction for future research rather than a primary conclusion of the current work. Longitudinal designs comparing novices and experts, combined with detailed documentation of training patterns, will be necessary to disentangle innate asymmetries, technique-specific requirements and practice-induced changes.

Despite these novel insights into bilateral differences in synergy timing and spinal drive during Tai Chi heel-kick, several limitations must be acknowledged. First, the sample size was limited to 12 elite athletes, which may constrain generalizability. Second, although NMF is the most widely used method for synergy extraction, its outcomes are sensitive to preprocessing parameters and model order selection (Torres-Oviedo and Ting, 2007), which may affect result stability. Third, this study focused only on single-execution analysis of the heel-kick movement; future research should incorporate longitudinal training interventions, fatigue or perturbation conditions, and multi-joint kinetic measurements to examine how lateral differences manifest under varying task demands. Extending such analyses to general and clinical populations will be essential to verify the consistency and functional significance of this lateralization phenomenon across groups. Moreover, integration with neuroimaging techniques may further elucidate the contributions of supraspinal centers to this process.

## Conclusion

This study revealed distinct leg-specific neural control strategies in elite Tai Chi athletes during the heel-kick movement. Although the left and right legs exhibited highly consistent muscle synergy composition—including hip extensor–dominant, knee extensor–dominant, ankle dorsiflexor, and postural stabilization synergies—their activation timing and spinal drive patterns differed. Specifically, the left leg demonstrated earlier synergy activation peaks and centers of activity (CoA), indicating a leading role in movement initiation and early-phase force generation. In contrast, the right leg synergies were more concentrated in the mid-to-late phase, suggesting a role in balance and recovery. Spinal statistical parametric mapping (SPM) further showed differential segmental drive within L2–S2: right-leg activation was more focused in L4–S1, whereas left-leg activation was distributed more broadly. These findings suggest that execution of complex Tai Chi lower-limb movements relies on a neural control strategy characterized by “conserved structure with differentiated timing.” The present study not only deepens the understanding of neuromuscular mechanisms underlying Tai Chi performance but also provides novel theoretical and practical implications for optimizing athletic training and guiding rehabilitation interventions.

## Data Availability

The raw data supporting the conclusions of this article will be made available by the authors, without undue reservation.
